# DISCOVER trial– Distal resection of the pancreas with or without coverage of the pancreatic remnant: study protocol of a randomised controlled trial

**DOI:** 10.1186/1745-6215-14-430

**Published:** 2013-12-14

**Authors:** Matthias Hassenpflug, Thomas Bruckner, Philip Knebel, Markus K Diener, Markus W Büchler, Jens Werner

**Affiliations:** 1 Department of General, Visceral and Transplantation Surgery, University of Heidelberg, Im Neuenheimer Feld 110, 69120 Heidelberg, Germany; 2 Institute of Medical Biometry and Informatics, University of Heidelberg, Im Neuenheimer Feld 305, 69120 Heidelberg, Germany; 3 Clinical Study Centre, Department of General, Visceral and Transplantation Surgery, University of Heidelberg, Im Neuenheimer Feld 110, 69120 Heidelberg, Germany

**Keywords:** Coverage procedure, Distal pancreatectomy, Falciform ligament, Pancreatic fistula

## Abstract

**Background:**

Distal pancreatectomy for benign and malignant tumours is the second most common surgical procedure on the pancreas. Postoperative pancreatic fistulas (POPF) represent the most significant clinical complication, causing prolongation of hospital stay and the need for additional diagnostic and therapeutic procedures. Although various techniques for preventing POPF have been evaluated, to date, there is no available technique that ensures closure of the pancreatic remnant.

**Methods/Design:**

DISCOVER will aim to investigate differences in the postoperative course after a distal pancreatectomy comparing the standard surgical technique with an alternative technique that provides additional coverage of the pancreatic remnant by the falciform ligament. The primary endpoint of this trial will be the rate of POPF. As secondary endpoints, incidence of postoperative morbidity and mortality, length of hospital stay, and quality of life will be assessed.

DISCOVER is a single-centre, randomised, controlled surgical trial. For statistical analysis, a binary logistic regression model will be used. With a level of significance of 5% and a power of 80%, a sample size of 75 patients per group has been identified as necessary.

**Discussion:**

The findings of this trial will help to evaluate the usefulness of the coverage procedure at reducing the rate of POPF. The results could influence the standard procedure for remnant closure after distal pancreatectomy.

**Trial-registration:**

Clinical trials register (DRKS-ID: DRKS00000546)

## Background

A distal pancreatectomy is generally performed in cases of benign and malignant tumours of the pancreatic body or tail. Today, even advanced tumours with infiltration into neighbouring organs can be resected with good long-term outcome. Increasing numbers of diagnosed cystic lesions of the pancreas are a further reason for the increased need for distal pancreatectomies [1,2]. Despite low mortality rates in high-volume centres, the morbidity of this operation remains high, at up to 64% [3].

The most common morbidity following a distal pancreatectomy is postoperative pancreatic fistula (POPF). Insufficient healing of the resection margin causes leaking of aggressive pancreatic fluid into the abdominal cavity. Passive drainage tubes are routinely placed intra-abdominally in distal pancreatectomies. If any fluid from the abdominal cavity shows elevated levels of amylase on or after the third postoperative day, a POPF has to be assumed according to the consensus definition [4]. Most patients with a POPF are not limited by their physical condition and require no additional diagnostic or therapeutic steps; this condition is defined as a Grade A POPF. In these patients, the drainage tubes are removed slowly and in a stepwise manner during the following days or weeks, most often as an outpatient treatment. If they are not drained, these enzyme-rich intra-abdominal fluid collections often induce pain, lack of appetite, and vomiting, resulting in a prolonged hospital stay. Secondary bacterial infection of these collections can cause additional septic complications to varying degrees. Clinically impaired patients often need further therapeutic interventions such as nasogastric tubes, parenteral nutrition, antibiotics, and interventional drainage. Very rarely, angiography or further surgery is needed. Depending on the patient’s condition and the therapeutic interventions needed, the patient may be diagnosed with clinically relevant Grade B or Grade C POPF (Table 1).

**Table 1:** **Definition and clinical grading of** POPF **according to the international study group on pancreatic fistula**

**Definition**	**Volume of drainage fluid on or after.**
**Grade A**	Clinical condition is good; at most little changes in management are needed. Hospital stay is not delayed; condition is managed by slow removal of drains.
**Grade B**	Clinical condition is often good; peripancreatic collection may occur. Specific forms of therapy, such as parenteral nutrition and antibiotics in cases of infection, are often needed. Endoscopic stenting of the main pancreatic duct may be necessary for sufficient drainage. Usually, hospital stay is delayed or readmission is required.
**Grade C**	Clinical condition is poor and stability may be borderline. Major changes in clinical management as intensive care and invasive procedures such as CT-guided drainage, angiography, or re-operations may be needed.

To date, various techniques for closure of the pancreatic stump have been tried and evaluated, including hand-sewn suturing, stapler closure, pancreatic duct ligation, ultrasonic dissection, pancreatoenteric anastomoses, application of meshes, and sealing with fibrin glue [3], yet none of these techniques can ensure a secure closure. New techniques are needed to avoid this complication.

### Preliminary data

Ever since distal pancreatectomies were first performed, there has been a longstanding debate as to the most effective technique for closure of the pancreatic remnant. For a long time, several different techniques were practiced side-by-side without good evidence in favour of or against any of them [5,6]. In 2005, a systematic review and meta-analysis verified this lack of evidence. Due to compromised comparability because of non-standardised surgical procedures or varying definitions of a POPF, the rates ranged from 0% up to 60% [7]. The DISPACT trial compared the two most commonly used techniques (hand-sewn suturing vs. stapler closure) in a large randomised multicentre trial. In that trial, the detected POPF rate was 36%, demonstrating that secure primary closure of the pancreatic resection margin is far from assured with the current techniques [3].

Recent reports have highlighted the potential advantage of additional coverage of the pancreatic remnant with autologous serous peritoneal tissue [5,6,8-13]. A prospective series from our institution confirmed that reduced rates of POPF were associated with shorter hospital stays and reduced treatment costs [14]. Closure with autologous patches is not a new technique, but it has long been underused. In the 1970s, peritoneal and pleural patches were used to prevent leakages in anastomoses after oesophageal and colorectal resections [15,16]. The falciform patch was initially used in the closure of duodenal and gastric perforations in high-risk patients [16]. In 2006, its use in the context of distal pancreatectomies was first described when skeletonised vessels were wrapped with a falciform patch to protect them from erosion bleeding due to POPF [17]. Iannitti et al. introduced the use of the falciform patch to cover pancreatic anastomoses in order to prevent POPF in pancreaticoduodenectomies and distal pancreatectomies in a retrospective series [10]. However, the results of recent studies highlighting this technique in distal pancreatectomies have to be interpreted with caution due to certain methodological drawbacks including mixed populations, inadequate or missing control groups, and non-uniform definitions of POPF.

## Study design

### Objectives and hypotheses

DISCOVER will aim to compare the effectiveness of additional reinforcement of the pancreatic stump by means of a coverage technique for preventing a POPF compared to hand-sewn closure alone in distal pancreatectomies.

The following hypotheses will be tested:

H_0_: The risk of developing POPF is equal in both groups.

H_1_: The risk of developing POPF is different between the two groups.

### Study population and location

The study population for the DISCOVER trial will consist of patients undergoing primary elective open surgery for benign and malignant tumours, chronic pancreatitis, or pseudocysts of the pancreatic body or tail. Detailed eligibility criteria are listed in Table 2.

**Table 2:** Eligibility criteria

**Inclusion criteria**	**Exclusion criteria**
● Aged 18 years and over	● Current immunosuppressive therapy
● Disease of the pancreatic body or tail or involving this part of the gland and planned treatment consisting of elective open distal pancreatectomy	● Participation in another trial that might conflict with the endpoints of this trial
	● Pre- or intraoperative sign for obstruction of the pancreatic duct in the head of the gland
● Informed consent provided	● Lack of informed consent or compliance
	● Inability to follow the study-explanations
● Intraoperative: performance of a distal pancreatectomy	

This single-centre trial will be performed at a high-volume institution with experience in pancreatic surgery (Department of General, Visceral and Transplantation Surgery, University Hospital of Heidelberg, Germany). The clinical study centre of the surgical department (http://www.klinikum.uni-heidelberg.de/Willkommen.130058.0.html) will conduct the DISCOVER trial.

### Sample size calculation

The sample size calculation is based on the expected rate of POPF after distal pancreatectomy with and without additional coverage of the pancreatic remnant. The expected POPF rate of 16% in the experimental group is based on the results of all available studies [5,6,8-10] in which coverage of the remnant was performed and the internationally accepted definition of POPF was used (according to ISGPF [4]; Table 1). The POPF rate in the control group is expected to be 36% based on the 30-day follow-up results of the DISPACT trial in which no group received additional coverage. With alpha = 5% and beta = 20%, a sample size of n = 75 per group is necessary to detect a difference between the two groups when the χ^2^ test (two-sided analysis) is used. It can be expected that including covariates of prognostic importance (age, BMI, and extent of resection) in the logistic regression model that is applied in confirmatory analysis will increase the power as compared to that of the χ^2^ test.

### Ethics, study registration and consent

Before the start of recruitment, this trial was approved by the independent ethics committee of the University of Heidelberg and registered at the German Clinical Trials Register (DRKS-ID: DRKS00000546). The DISCOVER trial will be conducted in the context of Good Clinical Practice and in accordance with the Declaration of Helsinki.

All patients assigned for distal pancreatectomy at the Department of General, Visceral and Transplantation Surgery, University of Heidelberg, will be screened for eligibility on the day before the operation. During this preoperative visit, patients will be informed about the clinical problem of POPF, the timeline of the DISCOVER trial and the possible risks and benefits of participation before they will be asked to give their written informed consent.

### Randomisation and the intention-to-treat principle

Patients will be randomised intraoperatively once the surgical decision to perform a distal pancreatectomy has been made. Block randomisation was chosen and a random list was generated using the PROC PLAN feature of SAS software™ (Cary, NC, USA). The randomisation process will be paper-based (using sequentially numbered opaque envelopes) and performed by members of the study centre at our surgical department. If a distal pancreatectomy will be performed but the randomised procedure is not fulfilled, the patient will stay in the allocated group for analysis, according to the intention-to-treat principle. If the surgical procedure of a distal pancreatectomy is not accomplished, e.g., because of inoperability or the need for total pancreatectomy, the patient will be excluded from final analysis.

### Study treatment

#### Standardised surgical approach (control group)

The type of abdominal incision (longitudinal or transverse laparotomy) will be determined by the surgeon performing the procedure. After exploration of the abdominal cavity, the pancreas will be revealed and transsected by scalpel (fish-mouth technique) or by stapler. The pancreatic duct will be identified and closed by crossing stitches. The dorsal and ventral edges of the resection margin will be adapted using single stitches. Further manipulation of the pancreatic remnant such as use of fibrin glue or reinforcement with meshes will not be allowed.

The decision to perform an additional splenectomy or cholecystectomy is up to the surgeon’s preference. Further abdominal organ resections (e.g., multivisceral resection), as well as resection of vessels, do not conflict with the protocol.

Photodocumentation of the pancreatic stump should be performed before closure of the abdomen to document the type of stump closure. Furthermore, members of the clinical study centre will make unannounced visits during operations to observe the surgical closure technique used on the pancreas.

Just before abdominal wall closure, two passive drainage tubes will be placed at the pancreatic remnant for percutaneous drainage.

#### Experimental group

**Figure 1:**
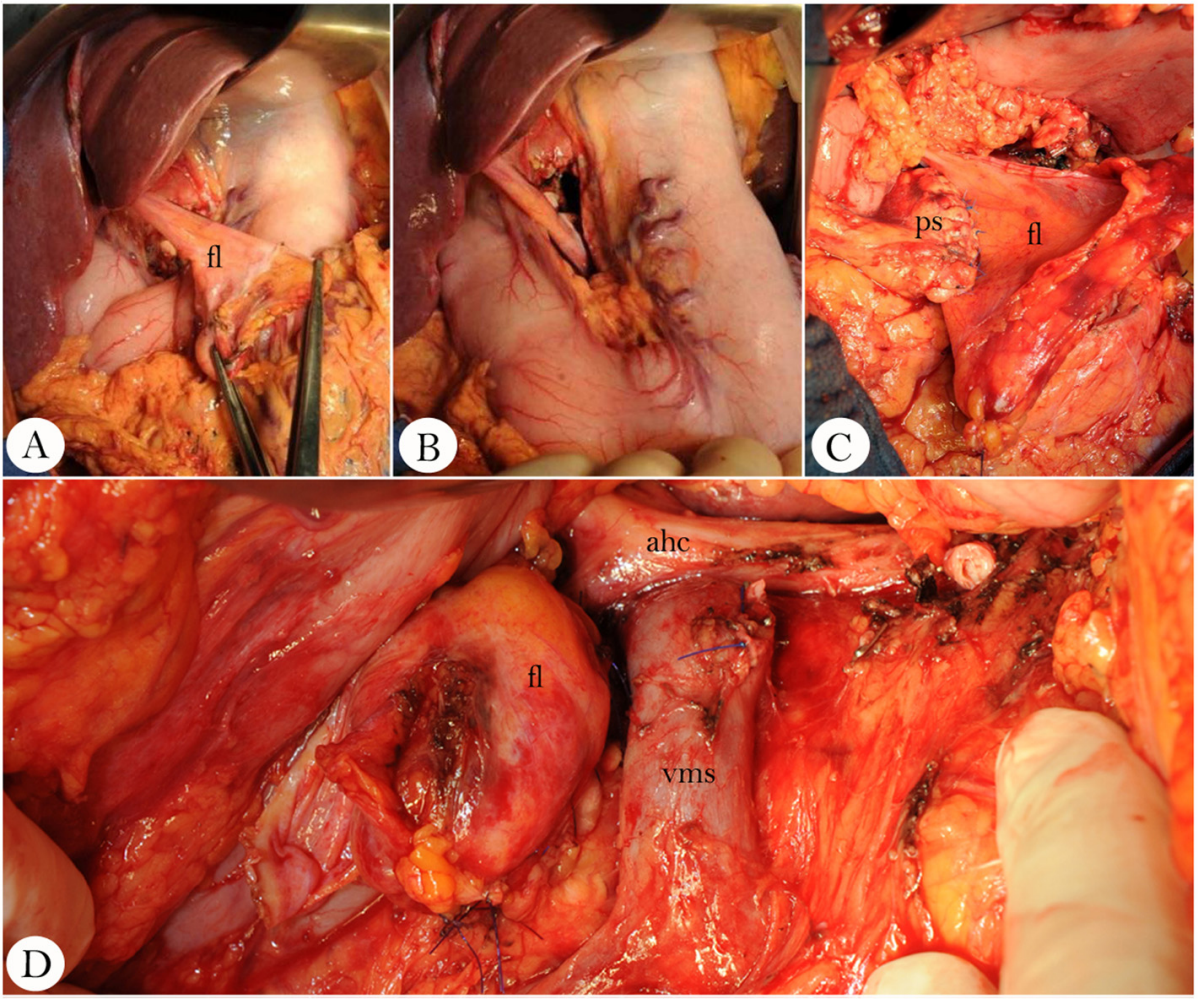
**Coverage procedure following distal pancreatectomy. (A)** The falciform ligament (fl) is separated from the abdominal wall **(B)** and pulled through the minor omentum. **(C + D)** Subsequently, a dorsal and ventral suture line (4–0 or 5–0 PDS) is used to fix the serosal surface of the ligament to the closed pancreatic stump (ps). vms = vena mesenterica superior; ahc = arteria hepatica communis.

In patients randomly assigned to receive the coverage procedure, the falciform ligament will be separated from the abdominal wall and pulled through the minor omentum to the resection margin of the pancreas and will be fixed to the ventral and dorsal surfaces of the gland by single stitches or running suture, to cover the complete closed margin (Figure 1).

If the falciform ligament cannot be used because of previous operations or for technical reasons, the gastric or jejunal wall can be used without opening of the intestinal lumen for the coverage procedure.

### Safety aspects of the coverage procedure

The falciform ligament and the ligamentum teres hepatis contained within it, which have no functional purpose in adults, are sutured to the pancreatic remnant by single stitches or running suture. A potentially elevated risk of developing delayed gastric emptying in patients with the falciform patch, as described in two reports cannot be excluded [5,8], but no additional complications associated with this interventional procedure have been described in the literature. Following the existing literature, it is expected that the coverage procedure will not extend operation time or increase blood loss, although both of these potential outcomes will be assessed as secondary endpoints within the trial [8,14].

### Adverse events and serious adverse events: accessing and reporting

An adverse event (AE) is any sign or symptom that impairs the patient’s well-being. A serious adverse event (SAE) is any AE that is life-threatening, prolongs hospital stay, results in persisting disability, or leads to death during the period of observation. AEs and SAEs will be recorded by means of daily postoperative ward visits until discharge and a telephone interview on postoperative day 40, performed by members of the study team (see below). All AEs of clinical relevance and all SAEs will be documented in the case report form. The trial investigator must be informed within 24 hours about SAEs that occur during hospital stay. The presence of a causal relationship between AEs or SAEs and the trial intervention will be judged by the investigator.

### Postoperative data collection

Daily visits with study patients on all wards will be performed by clinical investigators and study nurses attached to the clinical study centre in order to collect information on the primary and secondary outcome parameters and to identify any AEs or SAEs. All surgeons will have to describe the closure technique that they performed on the pancreas on a standardised form that will be sent to them by email on the day of the index operation to record whether patients were treated according to the study protocol and were assigned to their groups truly at random. After discharge, all medical documents will be reviewed for assessment of the endpoints.

The DISPACT trial demonstrated an increased rate of POPF (30% to 36%) between day 7 and day 30 because this is when re-admission due to the need for further treatment occurred. To ensure the accurate assessment of our endpoints, DISCOVER patients will be called by phone at least 40 days after the operation to record postoperative development.

All patients will be invited to be seen at the outpatient clinic (European Pancreas Centre, Department of General, Visceral and Transplantation Surgery, Heidelberg) three months after their operation for regular follow-up consisting of oral and physical examination and blood tests. CT or MR imaging will be performed for regular follow-up, independent from this trial. The results will be used for postoperative data collection. Our flowchart summarises the data collection process with regard to timing and data type (Table 3).

**Table 3:** Flowchart of the DISCOVER trial –course of examinations

**Visit**	**1**	**2**	**3**	**4**	**5**	**6**
	**Screening visit**	**Day of index OP**	**Daily postoperative ward visits**	**Day of discharge**	**Day 40 post OP (by phone)**	**3 months post-OP (optional)**
Past and current medical history	X					
Informed consent	X					
Physical examination and personal data (e.g., height, weight, age)	X					
Intraoperative randomisation (additional coverage yes/no)		X				
Basic study-related examination (to access endpoints)		X	X	X	X	X
Quality of life (EQ-5D questionnaire)	X			X	X	
AE, SAE		X	X	X	X	X
Drainage parameters (enzyme levels)			X	X		
Survival		X	X	X	X	X

### Primary and secondary endpoints

#### Primary endpoint

The primary endpoint of this trial is the rate of occurrence of POPF, as defined by the International Study Group on Pancreatic Fistula (ISGPF) [4]. A POPF is defined as the abdominal secretion of any measureable volume with amylase content greater than three times the upper normal value of serum amylase according to the centre-specific lab standards. Grading of a POPF mainly depends on its impact on clinical management. The need for CT-guided drainage shifts a POPF to Grade C since this is an invasive procedure (Table 1).

#### Secondary endpoints

Secondary endpoints will be postoperative morbidity including wound infection, intra-abdominal collection, delayed gastric emptying [18], postpancreatectomy haemorrhage [19], abdominal rupture, operation time, operative or interventional revisions, in-hospital mortality, duration of intensive care and hospital stay, need for readmission, and quality of life before operation, before discharge and at least 40 days after index operation according to the EQ-5D questionnaire (http://www.euroqol.org) (Table 4). The questionnaire that will be administered 40 days after the operation will be paper-based and sent to patients by post.

**Table 4:** Summary and definitions of secondary outcomes

**Outcome parameter**	**Definition**
**Wound infection**	Surgical site infection associated with laparotomy that develops during the hospital stay
**Intra-abdominal collection**	Fluid collections in the surgical site with or without signs of infection, usually shown in CT-scans
**Delayed gastric emptying (DGE)**	Inability to tolerate solid food with prolonged need for nasogastric tube for at least four days or nasogastric re-intubation after POD 3; grading of DGE depends on its impact on clinical course and management; three grades are used for differentiation [18]
**Postpancreatectomy haemorrhage (PPH)**	PPH is classified, according to time of onset (early vs. late), severity (mild vs. severe) and diagnostic and therapeutic consequences (observation, transfusion, interventional, operative), into three grades [19]
**Abdominal rupture**	Dehiscence of abdominal closure with need for relaparotomy during 40 days after index operation
**Operation time**	Time from skin incision to closure of wound (minutes)
**Operative or interventional revisions**	All actions for diagnostic or therapeutic reasons that are related to an abnormal postoperative course are documented for analyses.
**In-hospital mortality**	Death of any cause during hospital stay
**Need for readmission**	Readmission to any hospital due to postoperative complications
**Quality of life**	Quality of life, accessed by using the EQ-5D questionnaire (EuroQol Group Foundation)

### Methods for avoiding bias

#### Minimizing systemic bias

Block randomisation will be used to ensure that patient groups are comparable. Randomisation will be performed by study nurses attached to the clinical study centre at the Department of Surgery at the University of Heidelberg. The decision of whether to perform a distal pancreatectomy or a pancreaticoduodenectomy cannot be made before abdominal exploration has been performed. Therefore, randomisation will be performed as soon as the decision on a resection strategy has been achieved by the surgeon. However, if patients are excluded after randomisation, e.g., because of a need for total pancreatectomy, the randomisation number will not be reused.

#### Minimizing treatment bias

Although the coverage procedure is a technically simple method and quick to perform, we predict that surgeons who are unfamiliar with it will experience a learning curve until they overcome their individual technical difficulties. At the trial institution, however, the coverage procedure has been performed since 2009, so all surgeons participating in the study are familiar with it.

#### Minimizing measurement bias

Detection and grading of primary and secondary endpoints will be based on records kept during the hospital stay. Members of the study team will not be involved in treatment decisions. Blinding is not needed since occurrence of POPF is an objective endpoint that cannot be influenced by the patient [20]. Blinding of the surgeon is not feasible.

### Statistical methods

#### Analysis

Each patient’s allocation to one of the two analysis populations will be defined prior to the analysis and will be documented in the statistical analysis plan. We will distinguish between the following subpopulations: all patients who were randomly assigned to one of the groups will be analysed in the full analysis set according to the intention-to-treat principle. The per protocol analysis set will include patients without major protocol deviations, while in the safety analysis set patients will be analysed according to treatment rather than randomly. During data review, deviations from the protocol will be assessed as “minor” or “major”. Major deviations from the protocol will lead to the exclusion of the patient from the per protocol analysis set.

#### Confirmatory analysis

The null hypothesis of the proposed trial assumes that the rate of POPF type A–C is equal in the two intervention groups. A binary logistic regression model will be applied in order to compare POPF-rates in the two groups, adjusting for age, BMI and extent of resection. Confirmatory analysis will be based primarily on the full analysis set, which is consistent with the intention-to-treat principle as it includes all patients as randomised. Missing values will be replaced by means of the ICA-r method described by Higgins et al. [18]. Additionally, sensitivity analyses will be performed according to alternative methods for dealing with missing data, such as complete case analysis. In the final analysis, c_alpha = 0.0087 (alpha = 0.05, two-sided) will be applied.

Concerning the secondary endpoints, exploratory data analysis will be performed calculating appropriate summary measures for the empirical distribution (mean, standard deviation, median, minimum and maximum for continuous variables, and frequencies and percentages for categorical variables) as well as descriptive two-sided *P* values. Sensitivity analyses will be conducted for the perprotocol population as well as for the appropriate subgroup (e.g., covering methods). The safety analysis will include calculation and comparison of frequencies and rates of complications and SAEs. Furthermore, statistical methods will be used to assess the quality of the data and the homogeneity of the intervention groups. All analyses will be performed using SAS version 9.1 or higher.

## Discussion

Distal pancreatectomies are performed for benign or malignant tumours in most cases. Despite the increasing caseload, the morbidity associated with this kind of operation remains high, mainly due to POPF, which occur in up to 60% of patients [7]. Additional diagnostic and therapeutic procedures are needed for these patients, leading to prolongation of their hospital stay, readmission, and an increase of treatment costs [14]. Furthermore, POPF can persist for up to six months. It can be assumed that quality of life is considerably impaired in fistula patients.

The DISCOVER trial will evaluate the technique of additional coverage of the pancreatic remnant and its effectiveness at reducing the rate of POPF after distal pancreatectomies. Using the ISGPF-definition of POPF will enable us to compare our results to those of other trials. The thesis that quality of life is impaired by POPF will be tested by means of a validated questionnaire (EQ-5D questionnaire; EuroQol Group Foundation).

If the use of the coverage procedure can be shown to reduce the rate of POPF, this would help to establish the coverage procedure as a standard technique after distal pancreatectomy.

## Trial status

The DISCOVER trial is currently still recruiting. The last patient is expected to be recruited toward the spring of 2014.

## Abbreviations

AE: Adverse event; DISCOVER: Distal resection of the pancreas with or without coverage of the pancreatic remnant; ISGPF: International study group on pancreatic fistula; POPF: Postoperative pancreatic fistula; SAE: Serious adverse event.

## Competing interests

The authors declare that they have no competing interests.

## Authors’ contributions

MH wrote most parts of this protocol. TB helped to develop the statistical design. PK and MKD integrated important quality-control aspects of the study into the protocol and helped to adjust the reviewed protocol. MWB and JW provided scientific input for the trial’s background and rationale. They also defined the patient collective and the specifications of the study intervention. All authors read and approved the final manuscript.
